# *N*-(3-Chloro­benzo­yl)-2-nitro­benzene­sulfonamide

**DOI:** 10.1107/S160053681200640X

**Published:** 2012-02-17

**Authors:** P. A. Suchetan, Sabine Foro, B. Thimme Gowda, M. Shet Prakash

**Affiliations:** a Department of Chemistry, Mangalore University, Mangalagangotri 574 199, Mangalore, India; b Institute of Materials Science, Darmstadt University of Technology, Petersenstrasse 23, D-64287 Darmstadt, Germany; c Department of Chemistry, University College of Science, Tumkur University, Tumkur 572 102, India

## Abstract

In the title compound, C_13_H_9_ClN_2_O_5_S, the N—C bond in the C—SO_2_—NH—C segment has a *gauche* torsion with respect to the S=O bonds. The conformation between the N—H bond and the *ortho*-nitro group in the sulfonyl benzene ring is *syn*, and that between the C=O and the *meta*-Cl atom in the benzoyl ring is *anti*. The mol­ecule is twisted at the S—N bond, with a torsion angle of 65.41 (38)°. The dihedral angle between the sulfonyl benzene ring and the –SO_2_—NH—C—O segment is 75.0 (1)°, and that between the sulfonyl and the benzoyl benzene ring is 89.1 (1)°. The crystal structure features inversion-related dimers linked by pairs of N—H⋯O(S) hydrogen bonds.

## Related literature

For our studies of the effects of substituents on the structures and other aspects of *N*-(ar­yl)-amides, see: Gowda *et al.* (1999 ▶, 2006 ▶); *N*-(ar­yl)-methane­sulfonamides, see: Gowda *et al.* (2007 ▶); *N*-(substitutedbenzo­yl)-aryl­sulfonamides, see: Suchetan *et al.* (2012 ▶); *N*-chloro­aryl­amides, see: Jyothi & Gowda (2004 ▶) and *N*-bromo­aryl­sulfonamides, see: Usha & Gowda (2006 ▶)..
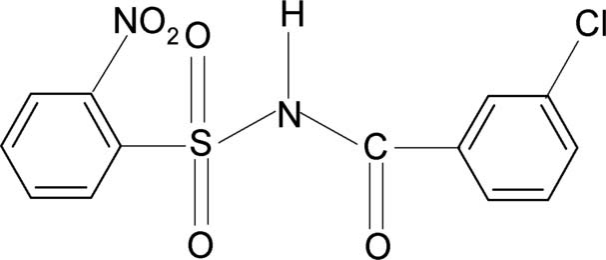


## Experimental

### 

#### Crystal data

C_13_H_9_ClN_2_O_5_S
*M* *_r_* = 340.73
Orthorhombic, 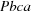

*a* = 12.2046 (8) Å
*b* = 12.6121 (9) Å
*c* = 18.433 (1) Å
*V* = 2837.3 (3) Å^3^
*Z* = 8
Mo *K*α radiation
μ = 0.44 mm^−1^
*T* = 293 K
0.28 × 0.28 × 0.08 mm

#### Data collection

Oxford Xcalibur diffractometer with Sapphire CCD detector
Absorption correction: multi-scan (*CrysAlis RED*; Oxford Diffraction, 2009 ▶) *T*_min_ = 0.886, *T*_max_ = 0.966
11298 measured reflections
2889 independent reflections
1911 reflections with *I* > 2σ(*I*)
*R*_int_ = 0.051

#### Refinement

*R*[*F* ^2^ > 2σ(*F* ^2^)] = 0.074
*wR*(*F* ^2^) = 0.147
*S* = 1.20
2889 reflections
202 parameters
1 restraint
H atoms treated by a mixture of independent and constrained refinement
Δρ_max_ = 0.34 e Å^−3^
Δρ_min_ = −0.29 e Å^−3^

### 

Data collection: *CrysAlis CCD* (Oxford Diffraction, 2009 ▶); cell refinement: *CrysAlis CCD*; data reduction: *CrysAlis RED* (Oxford Diffraction, 2009 ▶); program(s) used to solve structure: *SHELXS97* (Sheldrick, 2008 ▶); program(s) used to refine structure: *SHELXL97* (Sheldrick, 2008 ▶); molecular graphics: *PLATON* (Spek, 2009 ▶); software used to prepare material for publication: *SHELXL97*.

## Supplementary Material

Crystal structure: contains datablock(s) I, global. DOI: 10.1107/S160053681200640X/nc2268sup1.cif

Structure factors: contains datablock(s) I. DOI: 10.1107/S160053681200640X/nc2268Isup2.hkl

Supplementary material file. DOI: 10.1107/S160053681200640X/nc2268Isup3.cml

Additional supplementary materials:  crystallographic information; 3D view; checkCIF report

## Figures and Tables

**Table 1:** Hydrogen-bond geometry (Å, °)

*D*—H⋯*A*	*D*—H	H⋯*A*	*D*⋯*A*	*D*—H⋯*A*
N1—H1N⋯O1^i^	0.86 (2)	2.41 (3)	3.193 (4)	153 (4)

Symmetry code: (i) 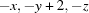.

**Fig. 1.**
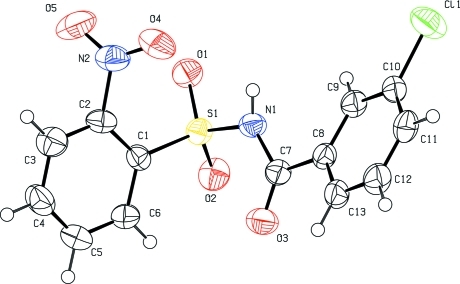
Molecular structure of the title compound, showing the atom-labelling scheme. Displacement ellipsoids are drawn at the 50% probability level.

**Fig. 2.**
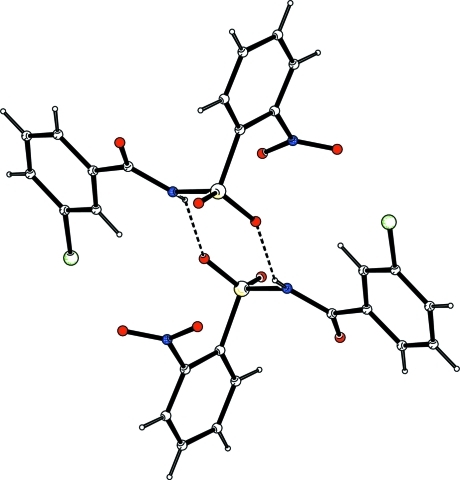
View of the inversion-related dimers linked by pairs of N—H···O(S) hydrogen bonds (Hydrogen bonding is shown as dashed lines)..

## supplementary crystallographic information

### Comment

As part of our studies on the substituent effects on the structures and other aspects of *N*-(aryl)-amides (Gowda *et al.*, 1999, 2006), *N*-(aryl)-methanesulfonamides (Gowda *et al.*, 2007), *N*-(substitutedbenzoyl)-arylsulfonamides (Suchetan *et al.*, 2012), *N*-chloroarylsulfonamides (Jyothi & Gowda, 2004) and *N*-bromoarylsulfonamides (Usha & Gowda, 2006), in the present work, the crystal structure of *N*-(3-chlorobenzoyl)-2-nitrobenzenesulfonamide has been determined (Fig.1).

The conformation between the N—H and C═O bonds in the C—SO_2_—NH—C(O) segment is *anti* and the N—C bond in the segment has *gauche* torsion with respect to the S═O bonds (Fig. 1), similar to that observed in *N*-(2-chlorobenzoyl)-2-nitrobenzenesulfonamide (I) (Suchetan *et al.*, 2012). In the title compound, the conformation between the N—H bond and the *ortho*-nitro group in the sulfonyl benzene ring is *syn*, similar to that observed in (I). Further, the conformation of the C═O is *anti* to the *meta*-Cl atom in the benzoyl ring, similar to that observed between the C═O and the *ortho*-Cl atom in (I).

The molecule is twisted at the S—N bond with the torsional angle of 65.41 (38)°, compared to the value of -59.68 (17)° in (I).

The dihedral angle between the sulfonyl benzene ring and the —SO_2_—NH—C—O segment is 75.0 (1)°, compared to the value of 77.5 (1)° in (I). Furthermore, the dihedral angle between the sulfonyl and the benzoyl benzene rings is 89.1 (1)°, compared to the value of 71.2 (1)° in (I).

In the crystal structure two molecules each are linked by pairs of intermolecular N—H···O (S) hydrogen bonds into dimers that are located around centers of inversion (Fig. 2 and Table 1).

### Experimental

The title compound was prepared by refluxing a mixture of 3-chlorobenzoic acid (0.02 mole), 2-nitrobenzenesulfonamide (0.02 mole) and excess phosphorous oxychloride for 3 h on a water bath. The resultant mixture was cooled and poured into crushed ice. The solid, *N*-(3-chlorobenzoyl)-2-nitrobenzenesulfonamide, obtained was filtered, washed thoroughly with water and then dissolved in sodium bicarbonate solution. The compound was later reprecipitated by acidifying the filtered solution with dilute HCl. It was filtered, dried and recrystallized.

Rod like colourless single crystals of the title compound used in X-ray diffraction studies were obtained by slow evaporation of the solvent from its toluene solution at room temperature.

### Refinement

The H atom of the NH group was located in a difference map and later restrained to N—H = 0.86 (2) %A. The other H atoms were positioned with idealized geometry using a riding model with C—H = 0.93 Å. All H atoms were refined with isotropic displacement parameters (set to 1.2 times of the *U*_eq_ of the parent atom).

### Crystal data

**Table:**

C_13_H_9_ClN_2_O_5_S	*F*(000) = 1392
*M**_r_* = 340.73	*D*_x_ = 1.595 Mg m^−^^3^
Orthorhombic, *P**b**c**a*	Mo *K*α radiation, λ = 0.71073 Å
Hall symbol: -P 2ac 2ab	Cell parameters from 1818 reflections
*a* = 12.2046 (8) Å	θ = 2.6–27.9°
*b* = 12.6121 (9) Å	µ = 0.44 mm^−^^1^
*c* = 18.433 (1) Å	*T* = 293 K
*V* = 2837.3 (3) Å^3^	Rod, colourless
*Z* = 8	0.28 × 0.28 × 0.08 mm

### Data collection

**Table:**

Oxford Xcalibur diffractometer with Sapphire CCD detector	2889 independent reflections
Radiation source: fine-focus sealed tube	1911 reflections with *I* > 2σ(*I*)
Graphite monochromator	*R*_int_ = 0.051
Rotation method data acquisition using ω and φ scans	θ_max_ = 26.4°, θ_min_ = 2.6°
Absorption correction: multi-scan (*CrysAlis RED*; Oxford Diffraction, 2009)	*h* = −13→15
*T*_min_ = 0.886, *T*_max_ = 0.966	*k* = −15→11
11298 measured reflections	*l* = −22→23

### Refinement

**Table:**

Refinement on *F*^2^	Primary atom site location: structure-invariant direct methods
Least-squares matrix: full	Secondary atom site location: difference Fourier map
*R*[*F*^2^ > 2σ(*F*^2^)] = 0.074	Hydrogen site location: inferred from neighbouring sites
*wR*(*F*^2^) = 0.147	H atoms treated by a mixture of independent and constrained refinement
*S* = 1.20	*w* = 1/[σ^2^(*F*_o_^2^) + (0.0362*P*)^2^ + 4.7309*P*] where *P* = (*F*_o_^2^ + 2*F*_c_^2^)/3
2889 reflections	(Δ/σ)_max_ < 0.001
202 parameters	Δρ_max_ = 0.34 e Å^−^^3^
1 restraint	Δρ_min_ = −0.29 e Å^−^^3^

### Special details

**Table:**

Experimental. CrysAlis RED (Oxford Diffraction, 2009) Empirical absorption correction using spherical harmonics, implemented in SCALE3 ABSPACK scaling algorithm.
Geometry. All e.s.d.'s (except the e.s.d. in the dihedral angle between two l.s. planes) are estimated using the full covariance matrix. The cell e.s.d.'s are taken into account individually in the estimation of e.s.d.'s in distances, angles and torsion angles; correlations between e.s.d.'s in cell parameters are only used when they are defined by crystal symmetry. An approximate (isotropic) treatment of cell e.s.d.'s is used for estimating e.s.d.'s involving l.s. planes.
Refinement. Refinement of *F*^2^ against ALL reflections. The weighted *R*-factor *wR* and goodness of fit *S* are based on *F*^2^, conventional *R*-factors *R* are based on *F*, with *F* set to zero for negative *F*^2^. The threshold expression of *F*^2^ > σ(*F*^2^) is used only for calculating *R*-factors(gt) *etc*. and is not relevant to the choice of reflections for refinement. *R*-factors based on *F*^2^ are statistically about twice as large as those based on *F*, and *R*-factors based on ALL data will be even larger.

### Fractional atomic coordinates and isotropic or equivalent isotropic displacement parameters (Å^2^)

**Table:**

	*x*	*y*	*z*	*U*_iso_*/*U*_eq_	
C1	−0.0112 (3)	0.7252 (3)	0.0676 (2)	0.0347 (9)	
C2	−0.0862 (3)	0.7030 (3)	0.0131 (2)	0.0402 (10)	
C3	−0.1369 (4)	0.6063 (4)	0.0079 (3)	0.0503 (12)	
H3	−0.1854	0.5924	−0.0298	0.060*	
C4	−0.1152 (4)	0.5307 (4)	0.0590 (3)	0.0552 (13)	
H4	−0.1498	0.4652	0.0563	0.066*	
C5	−0.0427 (4)	0.5506 (4)	0.1143 (3)	0.0537 (13)	
H5	−0.0286	0.4986	0.1488	0.064*	
C6	0.0092 (4)	0.6469 (3)	0.1188 (2)	0.0450 (11)	
H6	0.0582	0.6598	0.1564	0.054*	
C7	−0.0490 (3)	0.9234 (3)	0.1845 (2)	0.0389 (10)	
C8	−0.1239 (3)	1.0094 (3)	0.2089 (2)	0.0354 (9)	
C9	−0.1284 (3)	1.1061 (3)	0.1738 (2)	0.0390 (10)	
H9	−0.0812	1.1205	0.1354	0.047*	
C10	−0.2036 (4)	1.1812 (3)	0.1960 (2)	0.0400 (10)	
C11	−0.2730 (4)	1.1613 (4)	0.2528 (2)	0.0456 (11)	
H11	−0.3240	1.2119	0.2672	0.055*	
C12	−0.2663 (4)	1.0654 (4)	0.2884 (2)	0.0499 (12)	
H12	−0.3127	1.0518	0.3273	0.060*	
C13	−0.1921 (4)	0.9897 (3)	0.2670 (2)	0.0452 (11)	
H13	−0.1879	0.9254	0.2915	0.054*	
N1	−0.0153 (3)	0.9315 (3)	0.11212 (18)	0.0400 (9)	
H1N	−0.050 (3)	0.966 (3)	0.0794 (18)	0.048*	
N2	−0.1148 (3)	0.7825 (3)	−0.0428 (2)	0.0528 (10)	
O1	0.0765 (2)	0.8844 (2)	−0.00038 (15)	0.0478 (8)	
O2	0.1576 (2)	0.8265 (2)	0.11536 (16)	0.0514 (8)	
O3	−0.0201 (3)	0.8509 (2)	0.22210 (16)	0.0538 (8)	
O4	−0.1559 (3)	0.8640 (3)	−0.0218 (2)	0.0730 (11)	
O5	−0.0955 (3)	0.7592 (3)	−0.10577 (18)	0.0747 (11)	
Cl1	−0.20968 (12)	1.29932 (10)	0.14917 (7)	0.0697 (4)	
S1	0.06425 (8)	0.84464 (8)	0.07160 (6)	0.0381 (3)	

### Atomic displacement parameters (Å^2^)

**Table:**

	*U*^11^	*U*^22^	*U*^33^	*U*^12^	*U*^13^	*U*^23^
C1	0.036 (2)	0.030 (2)	0.038 (2)	0.0025 (18)	0.0033 (19)	0.0001 (18)
C2	0.037 (2)	0.044 (3)	0.040 (2)	0.007 (2)	0.0002 (19)	0.011 (2)
C3	0.042 (3)	0.054 (3)	0.055 (3)	−0.004 (2)	−0.008 (2)	0.004 (2)
C4	0.063 (3)	0.037 (3)	0.066 (3)	−0.011 (2)	−0.001 (3)	0.007 (2)
C5	0.068 (3)	0.041 (3)	0.052 (3)	0.005 (2)	−0.005 (3)	0.013 (2)
C6	0.052 (3)	0.043 (3)	0.040 (2)	0.007 (2)	−0.010 (2)	0.003 (2)
C7	0.044 (3)	0.037 (2)	0.035 (2)	−0.004 (2)	0.0004 (19)	0.0011 (19)
C8	0.039 (2)	0.036 (2)	0.031 (2)	−0.003 (2)	0.0033 (18)	−0.0040 (17)
C9	0.043 (3)	0.041 (2)	0.033 (2)	−0.004 (2)	0.0097 (19)	−0.0018 (18)
C10	0.049 (3)	0.033 (2)	0.037 (2)	−0.004 (2)	0.001 (2)	−0.0022 (18)
C11	0.047 (3)	0.048 (3)	0.042 (2)	0.002 (2)	0.009 (2)	−0.013 (2)
C12	0.060 (3)	0.050 (3)	0.040 (2)	−0.008 (3)	0.018 (2)	−0.005 (2)
C13	0.061 (3)	0.037 (2)	0.037 (2)	−0.006 (2)	0.009 (2)	−0.0048 (19)
N1	0.048 (2)	0.040 (2)	0.0329 (19)	0.0065 (18)	0.0043 (16)	0.0036 (16)
N2	0.049 (2)	0.057 (3)	0.053 (3)	−0.004 (2)	−0.012 (2)	0.016 (2)
O1	0.0518 (19)	0.0507 (18)	0.0411 (16)	−0.0011 (15)	0.0137 (14)	0.0022 (14)
O2	0.0366 (17)	0.060 (2)	0.0577 (19)	0.0028 (16)	−0.0076 (15)	−0.0045 (16)
O3	0.068 (2)	0.0485 (19)	0.0450 (17)	0.0083 (18)	0.0041 (16)	0.0094 (16)
O4	0.090 (3)	0.052 (2)	0.077 (3)	0.018 (2)	−0.007 (2)	0.0162 (19)
O5	0.087 (3)	0.098 (3)	0.0394 (19)	−0.011 (2)	−0.0108 (19)	0.0131 (19)
Cl1	0.0894 (11)	0.0479 (7)	0.0718 (9)	0.0188 (7)	0.0221 (7)	0.0146 (6)
S1	0.0379 (6)	0.0399 (6)	0.0365 (5)	0.0018 (5)	0.0035 (5)	−0.0007 (5)

### Geometric parameters (Å, º)

**Table:**

C1—C6	1.388 (5)	C8—C9	1.382 (5)
C1—C2	1.389 (6)	C9—C10	1.381 (6)
C1—S1	1.767 (4)	C9—H9	0.9300
C2—C3	1.371 (6)	C10—C11	1.369 (6)
C2—N2	1.479 (5)	C10—Cl1	1.723 (4)
C3—C4	1.366 (6)	C11—C12	1.378 (6)
C3—H3	0.9300	C11—H11	0.9300
C4—C5	1.373 (6)	C12—C13	1.374 (6)
C4—H4	0.9300	C12—H12	0.9300
C5—C6	1.372 (6)	C13—H13	0.9300
C5—H5	0.9300	N1—S1	1.643 (4)
C6—H6	0.9300	N1—H1N	0.857 (19)
C7—O3	1.201 (5)	N2—O4	1.207 (5)
C7—N1	1.399 (5)	N2—O5	1.221 (5)
C7—C8	1.489 (6)	O1—S1	1.426 (3)
C8—C13	1.379 (5)	O2—S1	1.414 (3)
			
C6—C1—C2	117.8 (4)	C8—C9—H9	120.2
C6—C1—S1	119.0 (3)	C11—C10—C9	120.8 (4)
C2—C1—S1	123.1 (3)	C11—C10—Cl1	121.0 (3)
C3—C2—C1	121.9 (4)	C9—C10—Cl1	118.2 (3)
C3—C2—N2	116.7 (4)	C10—C11—C12	119.2 (4)
C1—C2—N2	121.5 (4)	C10—C11—H11	120.4
C4—C3—C2	119.0 (4)	C12—C11—H11	120.4
C4—C3—H3	120.5	C13—C12—C11	120.8 (4)
C2—C3—H3	120.5	C13—C12—H12	119.6
C3—C4—C5	120.7 (4)	C11—C12—H12	119.6
C3—C4—H4	119.7	C12—C13—C8	119.7 (4)
C5—C4—H4	119.7	C12—C13—H13	120.2
C6—C5—C4	120.2 (4)	C8—C13—H13	120.2
C6—C5—H5	119.9	C7—N1—S1	123.9 (3)
C4—C5—H5	119.9	C7—N1—H1N	124 (3)
C5—C6—C1	120.4 (4)	S1—N1—H1N	108 (3)
C5—C6—H6	119.8	O4—N2—O5	126.1 (4)
C1—C6—H6	119.8	O4—N2—C2	116.9 (4)
O3—C7—N1	121.3 (4)	O5—N2—C2	117.0 (4)
O3—C7—C8	124.1 (4)	O2—S1—O1	120.19 (19)
N1—C7—C8	114.5 (4)	O2—S1—N1	108.94 (18)
C13—C8—C9	119.9 (4)	O1—S1—N1	104.51 (18)
C13—C8—C7	118.3 (4)	O2—S1—C1	107.76 (19)
C9—C8—C7	121.7 (4)	O1—S1—C1	108.41 (19)
C10—C9—C8	119.5 (4)	N1—S1—C1	106.23 (18)
C10—C9—H9	120.2		
			
C6—C1—C2—C3	−1.8 (6)	Cl1—C10—C11—C12	179.3 (3)
S1—C1—C2—C3	174.5 (3)	C10—C11—C12—C13	−0.7 (7)
C6—C1—C2—N2	178.6 (4)	C11—C12—C13—C8	−0.4 (7)
S1—C1—C2—N2	−5.0 (6)	C9—C8—C13—C12	1.7 (6)
C1—C2—C3—C4	1.8 (7)	C7—C8—C13—C12	−176.5 (4)
N2—C2—C3—C4	−178.7 (4)	O3—C7—N1—S1	0.6 (6)
C2—C3—C4—C5	−0.7 (7)	C8—C7—N1—S1	−178.1 (3)
C3—C4—C5—C6	−0.2 (7)	C3—C2—N2—O4	119.2 (5)
C4—C5—C6—C1	0.1 (7)	C1—C2—N2—O4	−61.2 (6)
C2—C1—C6—C5	0.9 (6)	C3—C2—N2—O5	−60.2 (6)
S1—C1—C6—C5	−175.6 (3)	C1—C2—N2—O5	119.4 (5)
O3—C7—C8—C13	−22.3 (6)	C7—N1—S1—O2	−50.4 (4)
N1—C7—C8—C13	156.3 (4)	C7—N1—S1—O1	179.9 (3)
O3—C7—C8—C9	159.5 (4)	C7—N1—S1—C1	65.4 (4)
N1—C7—C8—C9	−21.9 (6)	C6—C1—S1—O2	17.3 (4)
C13—C8—C9—C10	−1.7 (6)	C2—C1—S1—O2	−159.0 (3)
C7—C8—C9—C10	176.4 (4)	C6—C1—S1—O1	148.9 (3)
C8—C9—C10—C11	0.5 (6)	C2—C1—S1—O1	−27.4 (4)
C8—C9—C10—Cl1	−178.1 (3)	C6—C1—S1—N1	−99.3 (3)
C9—C10—C11—C12	0.7 (7)	C2—C1—S1—N1	84.4 (4)

### Hydrogen-bond geometry (Å, º)

**Table:**

*D*—H···*A*	*D*—H	H···*A*	*D*···*A*	*D*—H···*A*
N1—H1*N*···O1^i^	0.86 (2)	2.41 (3)	3.193 (4)	153 (4)

Symmetry code: (i) −*x*, −*y*+2, −*z*.
